# Case series on patients with primary central nervous system lymphoma: From clinical presentations to outcomes

**DOI:** 10.1002/ccr3.5447

**Published:** 2022-02-21

**Authors:** Kazem Anvari, James S. Welsh, Fatemeh Molaie

**Affiliations:** ^1^ 37552 Cancer Research Center Mashhad University of Medical Sciences Mashhad Iran; ^2^ Edward Hines Jr VA Hospital and Loyola University Chicago Stritch School of Medicine Chicago Illinois USA; ^3^ 37552 Mashhad University of Medical Sciences Mashhad Iran

**Keywords:** brain lymphoma, methotrexate, primary central nervous system lymphoma, whole‐brain radiotherapy

## Abstract

Primary central nervous system lymphoma (PCNSL) is a rare form of non‐Hodgkin lymphoma. In this report, we present 11 cases of PCNSL which were treated with high‐dose MTX and WBI with a localized radiation boost to the tumor bed.

## Graphical abstract

The definitive treatment of PCNSL is not clear, but most studies have indicated that high doses of methotrexate (MTX) followed by whole‐brain irradiation (WBI) can improve the outcome.

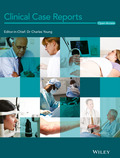

## 1 INTRODUCTION

Primary central nervous system lymphoma (PCNSL) is a rare form of non‐Hodgkin lymphoma arising from brain parenchyma, meninges, the spinal cord, or eyes without any systemic involvement. It accounts for up to 1% of all lymphomas and approximately 1%–4% of all CNS malignancies.^1^, ^2^, ^3^, ^4^, ^5^, ^6^, ^7^ In the immunocompromised patients, 16% of all primary CNS tumors can be reported as PCNSL.^8^ In fact, PCNSL in an HIV‐seropositive individual is an AIDS‐defining condition and as much as 6% of all AIDS patients might develop PCNSL.^9^ Nevertheless, PCNSL does occur in both immunocompromised and immunocompetent people with some distinct features.

Primary central nervous system lymphoma is commonly diagnosed in the elderly, with a median age of 65 years old.^2^, ^6^ The majority of PCNSL cases consist of diffuse large B‐cell lymphomas (DLBCLs). Other types include Burkitt's lymphoma, T‐cell lymphoma, and low‐grade malignant B‐cell lymphoma but these are extremely uncommon. It is not entirely clear yet if PCNSL truly arises from the CNS directly or if it originates as a systemic lymphoma which implants in the CNS by passing through the blood–brain barrier. Most cases of PCNSLs express B‐cell markers such as CD19, CD20, and CD79a. However, CD10 might be present in 10%–20%, and mutations in BCL6 and BCL2 might be presented in 60%–80% and 56%–93% of PCNSLs, respectively.^9^, ^10^, ^11^ Age and performance status have emerged as the most important prognostic factors.^1^, ^3^

The definitive treatment of PCNSL is unclear, but most studies have indicated that high‐dose (≥3.5 g/m^2^) intravenous methotrexate (MTX) followed by whole‐brain irradiation (WBI) can improve the outcome.^1^, ^4^ The role of the monoclonal antibody rituximab remains controversial. Although initially hoped to be of potential benefit, its high molecular weight raises the question of whether it adequately accumulates in the CNS to exert any significant anti‐lymphoma effect. The IELSG32 trial suggested that the addition of rituximab to high‐dose MTX and high dose‐cytarabine improved response rate, progression‐free survival, and overall survival.^12^, ^13^ In contrast, however, the results of the HOVON‐105/ALLG‐NHL‐24 study did not support the use of rituximab as a part of standard care for PCNSL.^14^, ^15^

Therefore, since PCNSL is a rare CNS disease and further research is required regarding its treatments, we aim here to present 11 cases of PCNSL and discuss their treatment strategies and their outcomes.

## 2 CASE SERIES

We present here 11 cases of PCNSL in which systemic lymphoma was ruled out through performing CT scan of neck, thorax, abdomen, and pelvic with or without intravenous contrast. Additionally, cerebrospinal fluid (CSF) analyses were negative for malignant cells in all cases.

### 2.1 Case number one

**FIGURE 1:**
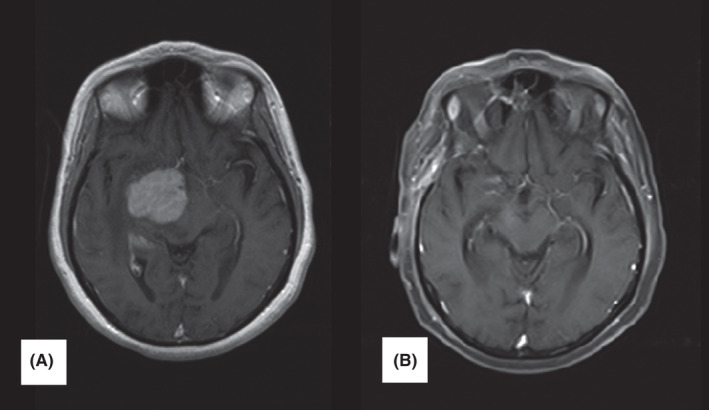
Case number 1. Post‐contrast axial brain MRI. (A) brain MRI before treatment: large intra‐axial mass in the medial right temporal lobe, thalamus, and basal ganglia with avid homogenous contrast enhancement, pressure on the lateral ventricle and midline shift. (B) brain MRI after 20 months follow‐up. An encephalomalacia focus in the right hemisphere without enhancement

A 44‐year‐old woman was referred to our department with weakness in left upper and lower limbs, urinary incontinency, dysarthria, and paresthesia of the left side of her face in the prior month. A brain MRI examination showed an enhancing mass in the right thalamic region and basal ganglia with mass effect in the right lateral ventricle. A brain biopsy was performed, and the pathological lesion was diagnosed as perivascular lymphocytic infiltration. She underwent four cycles of chemotherapy with high‐dose MTX (3.5 g/m^2^) but refrained from the two remaining prescribed cycles. Therefore, brain radiotherapy (RT) started and finished with a total dose of 36 Gy in 4 weeks (30.6 Gy to the whole brain followed by a localized boost to areas of gross involvement to 36 Gy). A brain MRI was performed 1 month later and showed post‐ radiation encephalopathy. After a 20‐month follow‐up, she has no signs or symptoms of recurrence (Figure 1).

### 2.2 Case number two

**FIGURE 2:**
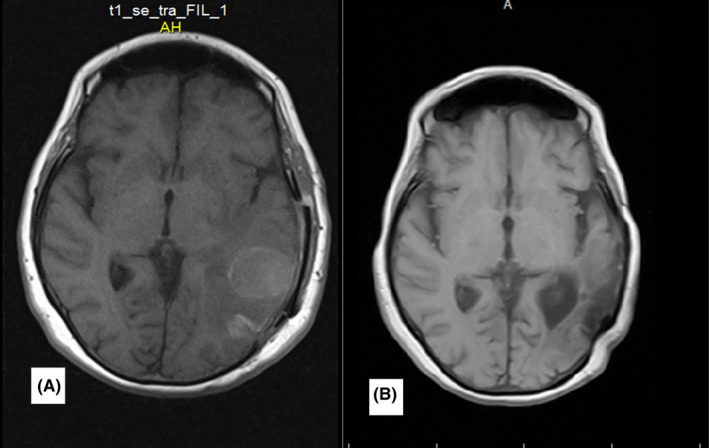
Case number 2. (A) brain MRI before treatment. Post‐contrast axial cut. A faint heterogeneous mass with mass effect in the left parietal lobe. (B) brain MRI after 30 months follow‐up. Axial brain MRI without contrast. Gliomalacia in the left parietal lobe

A 60‐year‐old woman with a history of headache and decrease in visual acuity had a faint heterogeneous mass with mass effect and edema in the left parietal lobe on her brain MRI. Biopsy was performed, and the diagnosis of malignant NHL (DLBL) was made. Immunohistochemistry (IHC) was as follows: CD20, LCA, and Ki67 were positive; CK, CD3, GFAP, and chromogranin were negative. After five cycles of high‐dose MTX (3 g/m^2^), the brain CT scan showed an unfavorable response. Thus, brain RT with a total dose of 43.2 Gy was performed in 5 weeks (30.6 Gy to the whole brain followed by a localized boost to 43.2 Gy). A brain MRI which was performed 1 month after finishing brain RT did not reveal any pathological findings. After a follow‐up of 33 months, she has not shown any signs of recurrence (Figure 2).

### 2.3 Case number three

A 42‐year‐old man was referred to our clinic with a history of headache, paresthesias of the right side of his face and the right hand. Brain MRI revealed an enhancing mass in the left parietal lobe, adjacent to the ventricles. Stereotactic biopsy was performed. Pathological findings showed PCNSL. He was treated with five cycles of high‐dose MTX (3.5 g/m^2^) chemotherapy and declined the 6th cycle. Follow‐up brain MRI revealed faint enhancement around the ventricles. Then, he underwent brain RT with a total dose of 36 Gy in 4 weeks (30.6 Gy to the whole brain followed by a localized boost to areas of gross involvement to 36 Gy). Another brain MRI had normal appearance 2 months after finishing RT. After 14 months of follow‐up, he remains without disease progression or sequelae of treatment.

### 2.4 Case number four

**FIGURE 3:**
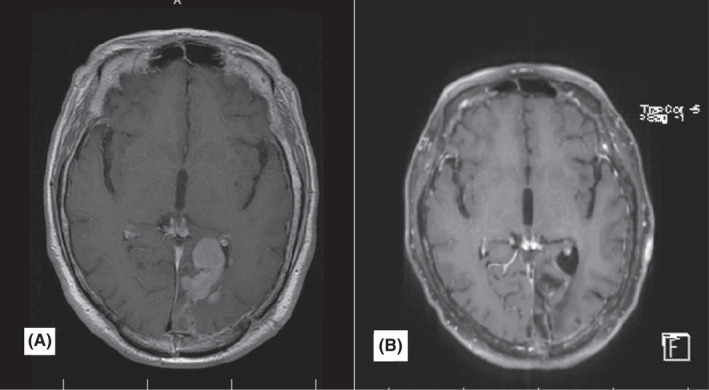
Case number 4. Post‐contrast axial brain MRI (A) brain MRI before treatment. Large mass in the left parietal lobe with avid non‐homogenous contrast enhancement. (B) after a follow‐up of 32 months. Gliomalacia in the left parietal lobe

A 60‐year‐old man with a history of headache and decreased visual acuity of the right eye was referred to us and a brain CT scan was performed, which revealed hypodense foci with edema and mass effect in the left parietal lobe. A biopsy was performed, and the diagnosis of PCNLS was made. He received two cycles of high‐dose MTX (3 g/m^2^) chemotherapy; a mid‐chemotherapy brain MRI was performed which was suggestive of a partial response. He then received three more courses of high‐dose MTX, followed by brain RT with a total dose of 39.6 Gy in 5 weeks (with a 8.4 Gy boost dose at 30.6 Gy). After a follow‐up of 33 months, he has no signs of recurrence (Figure 3).

### 2.5 Case number five

A 55‐year‐old man with amnesia was referred to our clinic with a brain MRI showing an enhancing mass with a solid and cystic component in the corpus callosum tail with vasogenic edema. Stereotactic biopsy and IHC confirmed CNS lymphoma. IHC showed: CD20 and LCA positive, CK and GFAP negative. He received five cycles of high‐dose MTX (3 g/m^2^) chemotherapy. After the 5th cycle, he developed tonic‐clonic seizures. He underwent brain RT with a total dose of 39.6 Gy in 5 weeks (localized after 30.6 Gy). A brain MRI after RT revealed encephalopathy in the right parietal lobe without recurrence. After 26 months of follow‐up, he bears no signs of recurrence.

### 2.6 Case number six

**FIGURE 4:**
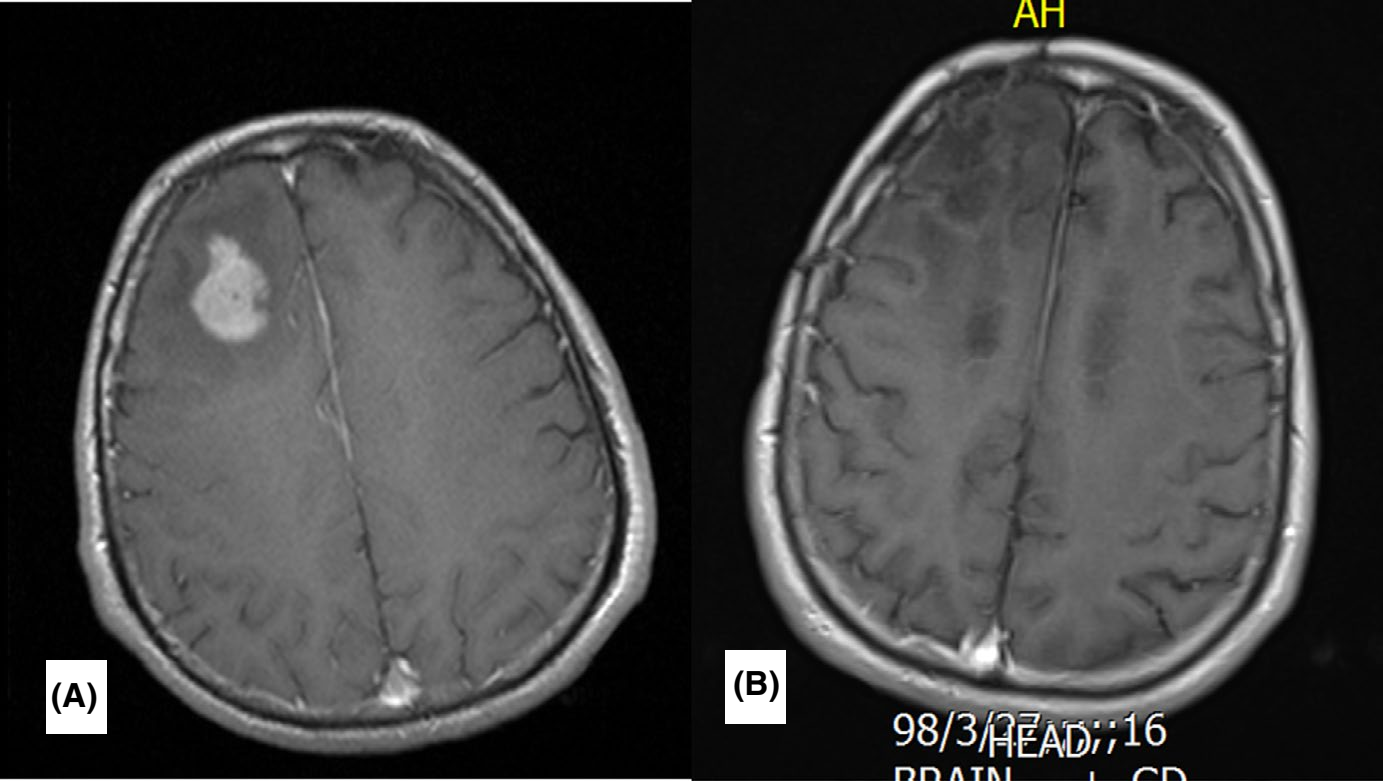
Post‐contrast axial brain MRI (A) brain MRI before treatment. An enhancing mass is evident in the right frontal lobe. (B) After 9 months of follow‐up, gliomalacia is seen in the right frontal lobe

A 54‐year‐old man with a history of headache and left‐side hemiparesis and seizure had an enhancing mass in his right frontal lobe with mass effect on his brain MRI. The biopsy of brain mass and IHC confirmed PCNSL. IHC was described as CD20 and LCA were positive; CK and GFAP were negative. He received five cycles of high‐dose MTX (3.5 g/m^2^) chemotherapy. Then, he underwent 36 Gy brain RT in 4 weeks (localized after 30.6 Gy). The brain MRI after treatment revealed hydrocephaly only, but he did not return for the following‐up appointment and expired 1 year following treatment (survival was 20 months; Figure 4).

### 2.7 Case number seven

A 63‐year‐old woman with a history of headache, depression, cognitive disorders, and urinary incontinency was referred to our clinic with a brain MRI revealing abnormal signal changes in the left frontotemporoparietal lobe with a considerable amount of edema and mild mass effect over the midline shifting structure. Biopsy of the lesion and IHC confirmed PCNSL. IHC showed that CD20 and LCA were positive; CK and GFAP were negative. She received four cycles of high‐dose MTX (3 g/m^2^) chemotherapy. After the 3rd cycle, her brain MRI revealed a complete response, but unfortunately, after the 4th cycle, she passed away due to pancytopenia and fever.

### 2.8 Case number eight

A 37‐year‐old woman with a history of headache, vertigo, and lower limb paraparesis had a brain MRI which showing two masses accompanied by edema in her cerebellum. A biopsy of brain mass and IHC confirmed brain lymphoma. IHC revealed that CD20 and LCA were positive; CK, EMA, CD3, HMB45, and NSE were negative. She received six cycles of high‐dose MTX (3.5 g/m^2^) and then underwent brain RT with a total dose of 30.6 Gy in 4 weeks. The brain MRI following RT revealed postoperative changes in the left hemisphere of the cerebellum without recurrence. After a follow‐up of 33 months, she has no signs of recurrence.

### 2.9 Case number nine

A 42‐year‐old woman with a history of amnesia and decrease in visual acuity was referred to the Department of oncology. Her brain MRI revealed two foci with enhancement and edema, one in temproparietooccipital lobe and the other one in the right ventricle. A biopsy of the brain tumor confirmed brain lymphoma. IHC for CD20 was positive. She received six cycles of high‐dose MTX (3 g/m^2^) chemotherapy. The brain MRI at the end of chemotherapy revealed a complete response. She then underwent brain RT to a total dose of 36 Gy in 4 weeks. At a follow‐up of 42 months, she still feels well and remains without evidence of disease.

### 2.10 Case number ten

**FIGURE 5:**
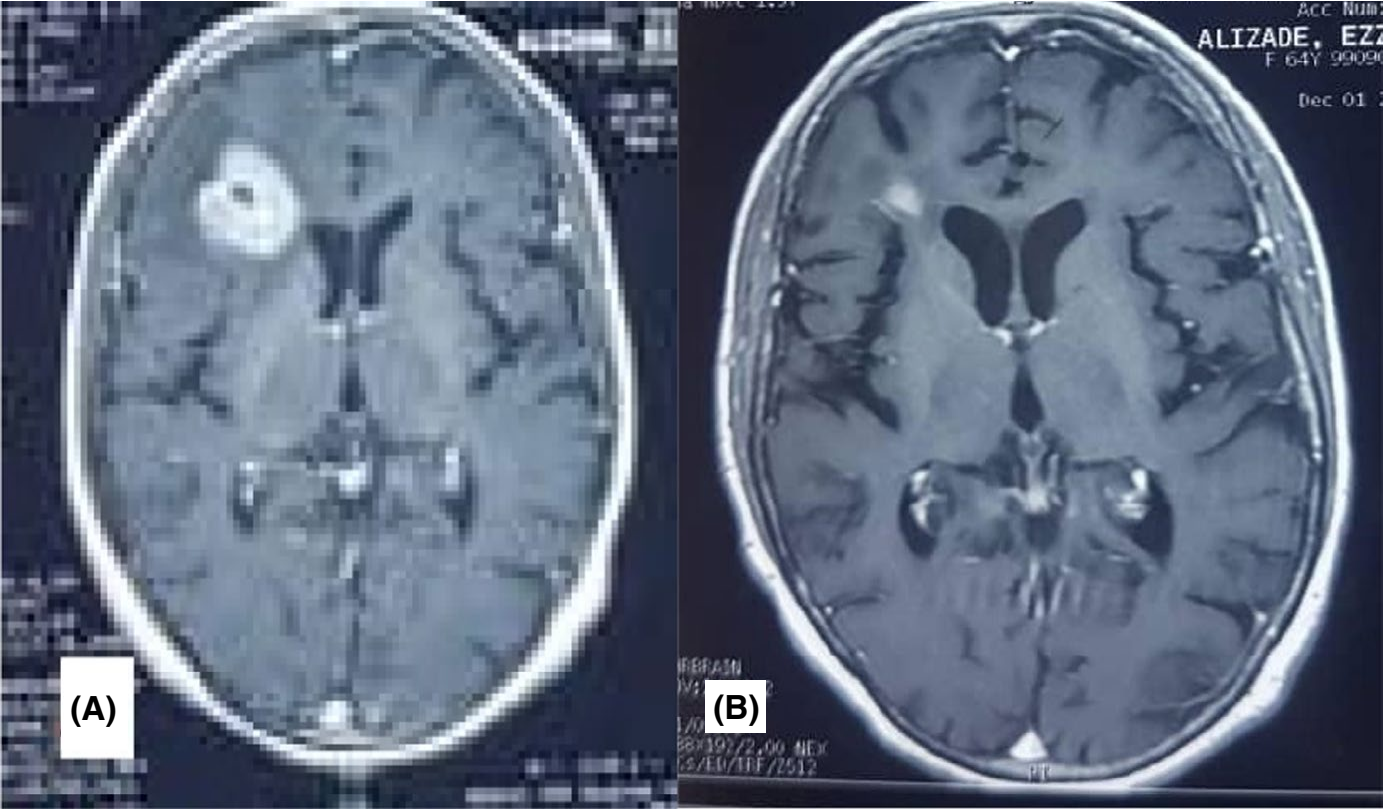
Post‐contrast axial brain MRI. (A) brain MRI before treatment. An enhancing lesion measuring 3 cm is present in the right frontal lobe. (B) 3 months after treatment. A subcentimeter enhancing lesion is visible in right frontal lobe

A 64‐year‐old woman with a history of blurred vision and a brain MRI that revealed an enhancing lesion measuring 3 cm in the right frontal lobe underwent stereotactic brain biopsy, and the diagnosis was DLBL. Chemotherapy with high‐dose MTX (3.5 g/m^2^) and rituximab (375 mg/m^2^) was given for three cycles. She had a partial response based on brain MRI so treatment was continued, but after the 6th cycle, her symptoms started to progress, and the chemotherapy protocol changed to rituximab plus cytarabine. The brain MRI after completing all chemotherapy revealed an enhancing lesion with a size of 3 cm plus edema in the frontal lobe. Thus, brain RT with a total dose of 30.6 Gy whole brain +14.4 Gy local boost was performed for her. She also received adjuvant temozolamide for 12 cycles. The brain MRI after treatment showed a subcentimeter lesion, and she remains with no evidence of progression after a follow‐up of 15 months (Figure 5).

### 2.11 Case number eleven

A 57‐year‐old woman with a history of left‐side hemiparesis and dysarthria had an enhancing mass with extensive edema in the right temporal lobe on brain MRI. Biopsy of the lesion and IHC confirmed brain lymphoma. IHC staining showed CD20 and LCA were positive; CD3, CK, and GFAP were negative. She received two cycles of high‐dose MTX (3 g/m^2^), but she did not have improvement in symptoms. Therefore, brain RT was done with a total dose of 39.6 Gy (localized after 30.6 Gy) in 5 weeks. Dysarthria and hemiparesis showed improvement, and the brain MRI revealed meningeal thickening and subdural effusion. Then, she received six cycles of R‐CHOP chemotherapy. After the treatment, she did not return for follow‐up, and she passed away 9 months after the diagnosis of PCNLS (Table 1).

**TABLE 1:** Characteristics of patients with PCNSL

Patient	Age	Gender	Sign and symptoms	MRI finding	IHC	Treatment	Complications of treatment	OS	Final Statues
1	44 years	Female	Weakness in left limbs, urinary incontinency, dysarthria, and paresthesia of the left side of her face	Showed an enhancing mass in the right thalamic region and basal ganglia with mass effect in the right ventricle	–	Four cycles of 4.5 g/m^2^ MTX plus brain RT with total dose of 36 Gy	Insomnia, headache, decreased appetite	20 months	Live
2	60 years	Female	of headache and decrease in visual acuity	a faint heterogeneous mass with mass effect and edema in the left parietal lobe	CD20, LCA, Ki67+ and CK, CD3, GFAP, chromogranin −	Five cycles of 3 g/m^2^ MTX and then brain RT with total dose of 43.2 Gy	–	33 months	Live
3	42 years	Male	headache, paresthesia of the right side of his face as well as the right hand		–	Five cycles of 3.5 g/m^2^ MTX and then brain RT with total dose of 36 Gy	–	14 months	Live
4	60 years	Male	headache and decreased visual acuity of the right eye	a large mass in the left parietal lobe with avid non‐homogenous contrast enhancement	–	Five cycles of 3 g/m^2^ MTX and then brain RT with total dose of 39.6 Gy	–	33 months	Live
5	55 years	Male	amnesia	an enhancing mass with a solid and cystic component in corpus callosum tail and edema	CD20, LCA+, CK, GFAP−	Five cycles of 3 g/m^2^ MTX and then brain RT with a total dose of 39.6 Gy	–	26 months	Live
6	54 years	Male	headache and left‐side hemiparesis and seizure	an enhancing mass in his right frontal lobe with mass effect	CD20, LCA+, CK, GFAP−	Five cycles of 3.5 g/m^2^ MTX and then brain RT with total dose of 36 Gy	Cataract	20 months	Dead
7	63 years	Female	headache, depression, cognitive disorders, and urinary incontinency	abnormal signal changes in the left frontotemporoparietal lobe with a considerable amount of edema and mild mass effect over the midline shifting structure	CD20, LCA+, CK, GFAP−	Four cycles of high‐dose MTX (3 g/m^2^) chemotherapy	Rise of serum creatinine, oral mucositis, pancytopenia, and death	3 months	Dead^a^
8	37 years	Female	headache, vertigo, and lower limb paraparesis	two masses accompanied by edema in her cerebellum	CD20, LCA+CK, EMA, CD3, HMB45, NSE −	Six cycles of high‐dose MTX (3.5 g/m^2^) chemotherapy and then underwent WBI with a total dose of 30.6 Gy	Mild neurocognitive disorders	33 months	Live
9	42 years	Female	amnesia and decrease in visual acuity	two foci with enhancement and edema, one in temproparietooccipital lobe and the other one, in the right ventricle	CD20 +	Six cycles of high‐dose MTX (3 g/m^2^) chemotherapy and then underwent brain RT with a total dose of 42 Gy		42 months	Live
10	64 years	Female	blurred vision	an enhancing lesion with a size of 3 cm in the right frontal lobe	–	Six cycles of Chemotherapy with 3.5 g/m^2^ MTX and Rituximab (375 mg/m^2^), then Rituximab plus cytarabine, and then brain RT with a total dose of 30.6 Gy	Dizziness, Mild neurocognitive disorders	15 months	Live
11	57 years	Female	left‐side hemiparesis and dysarthria	an enhancing mass with extensive edema in the right temporal lobe	CD20, LCA+ CD3, CK GFAP−	Two cycles of 3 g/m^2^ MTX and then brain RT with total dose of 39.6 Gy, and after those Six cycles of R‐CHOP chemotherapy	Candidiasis of mouth, dizziness, weakness	9 months	Dead

^a^ During the chemotherapy course due to chemotherapy‐induced pancytopenia.

## 3 DISCUSSION

The most common symptoms of PCNSL include neurocognitive disorders.^16^ The most common symptoms observed in our patients were headache and visual disorders. In most studies, the median age at diagnosis is 65 years, but our patients were 37–63 years old, and as we know, younger patients with good performance status more likely to tolerate treatment with high‐dose methotrexate‐based chemotherapy with or without radiotherapy. Therefore, it is not unexpected that they will have a longer survival rate.

Primary central nervous system lymphoma in brain MRI reveals iso‐ or hypointense signals in T1‐weighted images, iso‐ or slightly hyperintense signals on T2‐ weighted images, hyperintense signals on FLAIR images, and plaque enhancement on gadolinium contrast‐enhanced images.^17^ It is typically located in the supratentorial region, and the most common areas of involvement include the cerebral hemispheres, basal ganglia, thalamus and corpus callosum.^18^ Eight of our patients had a tumor in the cerebral hemispheres, and the other involved sites were the thalamus, corpus callosum, and cerebellum. The majority of PCNSLs consist of diffuse large B‐cell lymphoma (DLBCL). Consistent with that observation, 11 of our patients were diagnosed with DLBCL.

The standard treatment for PCNSL is not clear. Surgical resection does not contribute to the management of PCNLS.^2^, ^6^, ^19^, ^20^ Moreover, temozolomide as recommended in glioma of brain has no proven role in the management of PCNSL.^21^ According to the majority of studies on PCNSL, chemotherapy based on high‐dose methotrexate (HD‐MTX) followed by WBRT is the most effective treatment of PCNSL but is accompanied by high neurotoxicity, especially in elderly populations.^19^, ^20^, ^22^, ^23^, ^24^, ^25^, ^26^ Therefore, there have been numerous studies aiming to reduce the risk of neurotoxicity with similar survival rates, such as HD‐MTX in combination with other types of chemotherapy (dexamethasone, etoposide, ifosfamide and carboplatin/cytarabine, thiotepa, rituximab/ cytarabine), low‐dose WBRT with tumor bed boost in combination with HD‐MTX, HD‐MTX with an autologous stem transplant, and HD‐MTX alone for patients older than 60 years old.^2^, ^20^, ^22^, ^23^, ^27^ The exact dose of MTX is not the same in all studies, and it can be administered at doses of 1–8 g/m^2^. In a phase 2 trial, HD‐MTX +cytarabine followed by WBRT was superior to HD‐MTX followed by WBRT. The IELSG, phase 2 trial compared three types of combination chemotherapy: 1) MTX + cytarabine; 2) MTX + cytarabine + rituximab, and 3) MTX + cytarabine + rituximab + thiotepa (MATRix). In this study, the combination of four drugs (arm 3, MATRix) was associated with improved tumor response.^2^

When PCNSL was treated using WBRT alone, the median overall survival rate was about 16 months but studies indicated survival can improve to more than 30 months with HD‐MTX followed by WBRT.^22^ The WBRT dose used for PCNSL is typically 30–50 Gy.^7^, ^28^ In a retrospective study conducted by Lee et al.^22^ HD‐MTX followed by WBRT with tumor bed boost had less neurotoxicity with comparable survival rates as a higher dose to the whole brain.

We used HD‐MTX with doses of 3–4.5 g/m^2^ for 4–6 cycles; we used cytarabine +HD‐MTX followed by brain RT in one case. The RT dose that we have used for most of our patients was 30.6 Gy as WBRT and a maximum dose of 14.4 Gy as a tumor bed boost (local planning target volume) with daily fractions of 1.8 Gy/day. Local PTV was defined by adding 0.5 cm margin to the clinical target volume (CTV), and the CTV was defined by a 1 cm margin to the post‐contrast enhancing lesion on the first brain MRI.

In general, the 5‐year survival rate of PCNSLs is about 33% with treatment. When not treated, the overall survival of patients with PCNSL is about 1.5 months.^29^ The survival rates of our patients ranged from 3 to 42 months with a median of 22.6 months. More follow‐up appointments are required for alive patients.

Studies have shown that high‐dose MTX in combination with WBRT improved the outcomes in adults.^1^, ^4^, ^6^ Our patients were treated using this protocol, and the majority had good survival. A tumor bed boost after WBRT in combination with high‐dose MTX can be as effective as high‐dose MTX in addition to WBRT with less neurotoxicity.^22^ Thus, we used this protocol for most of our patients and we found it yielded good responses, although further follow‐up appointments are still required. We must also keep this in mind that in patients aged 60 years or over, neurocognitive side effects are more prominent with WBRT, so there is a trend to use high‐dose MTX alone in these patients and the use of RT should be based on the relative benefits and complications.^30^

## CONFLICT OF INTEREST

Authors declare that they have no conflict of interest.

## AUTHOR CONTRIBUTIONS

K.A. and F.M. contributed in conception, design, and drafting of the manuscript. M.D contributed in data collection. K.A., F.M., and J.S.W. contributed in drafting of the manuscript. K.A. supervised the study. All authors approved the final version for submission.

## ETHICAL APPROVAL

The study was approved by Mashhad University of medical Sciences. The study conforms to recognized standards is of Declaration of Helsinki.

## CONSENT

The informed written consent forms were obtained from the patients.

## Data Availability

The data sets used and/or analyzed during the current study are available from the corresponding authors per request.
